# PD-L1 Expression Is Increased in a Subset of Basal Type Breast Cancer Cells

**DOI:** 10.1371/journal.pone.0088557

**Published:** 2014-02-14

**Authors:** Hatem Soliman, Farah Khalil, Scott Antonia

**Affiliations:** 1 Department of Women’s Oncology and Experimental Therapeutics, Moffitt Cancer Center, Tampa, Florida, United States of America; 2 Department of Anatomic Pathology, Moffitt Cancer Center, Tampa, Florida, United States of America; 3 Department of Thoracic Oncology and Immunology, Moffitt Cancer Center, Tampa, Florida, United States of America; University of Alabama at Birmingham, United States of America

## Abstract

**Background:**

Tumor cells express programmed death ligand 1 (PD-L1) and is a key immune evasion mechanism. PD-L1 expression in multiple breast cancer cell lines was evaluated to identify intrinsic differences that affect their potential for immune evasion.

**Methods:**

PD-L1 expression was analyzed in six breast cancer cell lines: AU565&MCF7 (luminal), BT20&HCC1143 (basal A), MDA231&HCC38 (basal B). Surface and intracellular PD-L1 expression +/− interferon γ for 48 hours was measured by flow cytometry. PD-L1 gene expression data for all breast cancer cell lines in the Comprehensive Cell Line Encyclopedia (CCLE) was analyzed. Correlation between PD-L1 levels and clinicopathologic parameters was analyzed within Oncomine datasets. A tissue microarray containing 61 invasive breast cancer primary tumor cores was stained for PD-L1 expression and analyzed.

**Results:**

Basal breast cancer cells constitutively express the highest levels of PD-L1. All cell lines increased PD-L1 expression with interferon γ, but basal B cells (MDA-231 and HCC38) demonstrated the largest increases. There were no differences in protein localization between cell lines. In the CCLE data, basal cell lines demonstrated higher mean PD-L1 expression compared to luminal cell lines. High PD-L1 expressing basal cell lines over-express genes involved in invasion, proliferation, and chemoresistance compared to low PD-L1 basal cell lines. High PD-L1 basal cell lines had lower expression of IRF2BP2 and higher STAT1 levels compared to low PD-L1 expressing cell lines. Within Oncomine datasets PDL1 mRNA levels were higher in basal type tumors. The TMA analysis demonstrated that lymph node positive cases had higher levels of PD-L1 protein expression compared to lymph node negative cases.

**Conclusions:**

Basal type breast cancer (especially basal B) express greater levels of PD-L1 constitutively and with IFN γ. High PD-L1 basal cells over-express genes involved in invasion, motility, and chemoresistance. Targeting PD-L1 may enhance eradication of aggressive breast cancer cells by the immune system.

## Introduction

Programmed cell death 1 ligand 1 (PD-L1, CD274, B7-H1) is encoded by the CD274 gene on chromosome nine under the control of an interferon regulatory factor 1 (IRF1) and Signal Transducer Activation of Transcription 1 (STAT1) response elements within its promoter. [1] PD-L1 is a 40kDa transmembrane protein that is expressed on a wide variety of normal tissues including natural killer cells, macrophages, myeloid dendritic cells, B cells, epithelial cells, and vascular endothelial cells. [2] Its normal physiologic role is to bind programmed death 1 receptors (PD-1) expressed on the surface of activated cytotoxic T cells. This binding causes inhibition of IL-2 production and T cell activation through reduced phosphorylation of ZAP70 and PKC θ. [3] The PD-1/PD-L1 interaction serves as an important regulatory check against an excessive adoptive immune response to antigens and autoimmunity. [4].

The expression of PD-L1 has been evaluated in a number of different tumor types including breast cancer.[5]–[7] Ghebeh et al. reported that PD-L1 expression was associated with a variety of adverse features such as higher grade, negative estrogen receptor status, and increased infiltration with T regulatory cells. [8], [9] Parsa et al published data showing activated PIK3CA signaling through PTEN loss resulted in high PD-L1 expression in gliomas. [10] The prior studies focused on the relationship between PD-L1 and traditional clinicopathologic breast cancer classifiers. Since our understanding of breast cancer biology has evolved with the advent of genomic classification schemes, it is imperative to understand how PD-L1 behaves within different genomic subtypes as well. This is especially important given the fact that while there is overlap between classical and genomic subtypes (i.e. triple negative and basal), they are not synonymous. We sought to explore this question further by looking at different breast cancer cell lines classified as luminal or basal. Furthermore, looking at the cancer cell lines without the influence of tumor stroma or immune infiltrates would provide additional information on intrinsic tumor cell properties that are associated with PD-L1 expression. This study identified a subset of basal breast cancer cells lines with much higher PD-L1 expression compared to other basal and luminal cell lines. Molecular and pathologic data suggests that these high PD-L1 expressing cells may behave in a more invasive and aggressive fashion. Not only would this data be informative for in vitro immunology assays that use breast cancer cell lines, but could be informative in breast cancer clinical research of PD-1/PD-L1 blockade as well.

## Methods

### Cell culture conditions

Assay ready cells were obtained from American Type Culture Collection (ATCC, Manassas VA) and are shown in Table 1.

**Table 1:** ATCC cell lines used in the flow cytometry analysis.

ATCC Breast Cancer Cell Line Panel				
CELL LINE	SUBTYPE	ER	PR	HER2
AU565	Luminal/HER2	−	−	+
MCF7	Luminal	+	+	−
BT-20	Basal A	−	−	−
HCC1143	Basal A	−	−	−
MDA-MB-231	Basal B	−	−	−
HCC38	Basal B	−	−	−

### Flow cytometry for PD-L1 expression, protein localization

Assay ready cell lines (ATCC) were treated with 10ng interferon gamma (IFN γ) (R&D) for 48 hours. After which they were harvested and counted. 20uL of anti-PD-L1 FITC (BD Bioscience) was added for 1 hour at 4C. Cells were washed and stained with PI (Invitrogen). Data acquisition was performed on a LSR II with FACSDiva software (BD Bioscience). Data analysis was performed using FloJo software (Tree Star). Mean fluorescence intensity (MFI) was determined by gating on live cells and subtracting background (isotype) MFI.

For intracellular flow cells were treated the same as above except they were stained with Live/dead violet fixable dye (Invitrogen) for 30 min prior to addition of PDL1 for surface staining. After surface stain cells were washed and fixed in 4% paraformaldahyde for 15 minutes. Washed and treated with Perm buffer (BD Bioscience) for 30 minutes then stained with 20 uL anti-PD-L1 for another hour at 4°C. Cells were then washed and flow was ran the same as above. Intracellular protein was calculated by subtracting the MFI of fully stained samples from surface only stained samples.

### Comprehensive cell line encyclopedia (CCLE) data analysis

The data was accessed at the following URL: http://www.broadinstitute.org/ccle/home. Using the GENE-E analysis tool on the website, the expression dataset of all breast cancer cell lines was downloaded. Two sided, unpaired T test was used to compare the mean Robust Multi-array Average (RMA) expression levels between basal and non-basal cell lines. The Gene Set Enrichment Analysis (GSEA) tool in the CCLE database was then used to compare differential genes expressed between two groups of high (BT20, CAL120, CAL851, HCC1954, HCC70, HS578T, MB-231,HDQ-P1, HCC1937,HCC38, MB-436, BT-549) versus low (DU4475, CAL51, HCC1143, HCC1187, HCC1569, HCC1395, HCC1500, HCC1599, HCC2157, MB-157, MB-468, HCC1806) PD-L1 expressing basal cell lines on Affymetrix U133+2 CEL file data. The groups were separated by the median PDL1 RMA expression level for the group. The cutoff used for enriched pathways within high PDL1 expressing cell lines was a false discovery rate of <25%, and the genes listed within the individual pathways are those which contributed the most to the enrichment score for each pathway listed. The listed individual genes were designated “core enriched” by the GSEA algorithm. Documentation regarding these criteria recommended by the GSEA tool is available online at http://www.broadinstitute.org/cancer/software/gsea/wiki/index.php/Main_Page. Mutation analysis was done by tabulating all the somatic mutations for the PD-L1 high and PD-L1 low basal cell lines documented in CCLE and looking for exclusive mutations in either the high or low group which had functional relevance. The STAT1 and IRF2BP2 gene expression analysis between high versus low PD-L1 groups used unpaired T tests with a two sided p value and descriptive statistics were generated along with 95% confidence intervals.

### Oncomine analysis

Oncomine (Compendia Bioscience, Ann Arbor, MI) was used for analysis and visualization of the Cancer Genome Atlas and Gluck annotated breast cancer datasets (http://www.oncomine.org). PD-L1 RNA expression levels were displayed using log2 median centered ratio boxplots for triple negative vs. non triple negative, PAM50 molecular subtypes, and nodal status (node − vs. node +).

### Tissue microarray PD-L1 analysis

A slide was cut from a pre-existing invasive ductal carcinoma TMA maintained by the Moffitt tissue core (breast 2B) with de-identified clinical information (age, TNM stage, histology, estrogen receptor status, progesterone receptor status, human epithelial growth receptor 2 status (HER2), grade) annotated for each of the 61 available cores. This work was conducted under the review and approval of the University of South Florida Institutional Review Board-Human Research Protection committee (FWA#00001669) under protocol number MCC16015. The informed consent requirement was waived as these were archival, de-identified, annotated tissue specimens from a pre-existing TMA maintained by the Moffitt Tissue Core. No patient specific protected health information was used in the analysis.

### Immunohistochemical staining

Slides were stained using a Ventana Discovery XT automated system (Ventana Medical Systems, Tucson, AZ) as per manufacturer’s protocol with proprietary reagents. Briefly, slides were deparaffinized on the automated system with EZ Prep solution (Ventana). Heat-induced antigen retrieval method was used in Cell Conditioning 1 (Ventana). The rabbit primary antibody that reacts to PD-L1, (#10084-R015, Sino Biological, China) was used at a 1∶200 concentration in Dako antibody diluent (Carpenteria, CA) and incubated for 60 min. The Ventana OmniMap Anti-Rabbit Secondary Antibody was used for 16 min. The detection system used was the Ventana ChromoMap kit and slides were then counterstained with hematoxylin. Slides were then dehydrated and coverslipped as per normal laboratory protocol. Scoring of PD-L1 was performed using digital image analysis software (Aperio Spectrum System) and positive pixel scoring algorithm version 9. The PD-L1 score used in the analysis was total positive pixel counts for PD-L1 within the core divided by the total pixel count multiplied by 100. A pathologist (FK) independently scored the tissue as a quality control on the image analysis scoring algorithm. Statistical analysis of the mean PD-L1 expression level (as a continuous variable) was compared between estrogen receptor (+/−), grade ½ vs. 3, and node status (N0/N1–3) using a nonparametric Kolmogorov-Smirnov test comparing cumulative distributions with Graphpad Prism 6 software. HER2 was not evaluated due to insufficient number of positive cases on the microarray for meaningful comparison.

## Results

### Flow cytometry analysis of PD-L1 expression across the six ATCC cell lines

**Figure 1:**
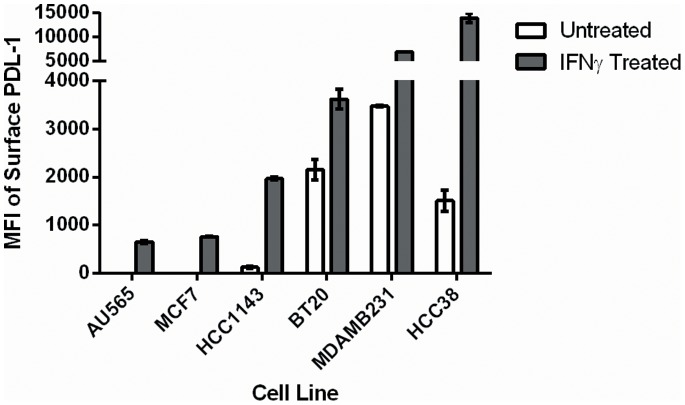
PD-L1 expression across the six different ATCC cell lines. Basal subtypes (especially basal B) demonstrated much greater constitutive PD-L1 expression than luminal subtypes. Treatment with IFNγ caused PD-L1 expression to increase in all cell lines but basal subtypes demonstrated much greater inducible levels of PD-L1.

As shown in Figure 1, there were considerable differences in both constitutive and IFN γ induced PD-L1 surface expression as measured by mean fluorescence intensity between the cell lines. The two luminal subtypes (MCF-7 and AU565) had the lowest levels of expression. The HCC1143 basal A cell line had low constitutive expression but significantly upregulated PD-L1 in response to IFN γ. BT20 had higher constitutive and inducible expression when compared to luminal subtypes. The highest constitutive expression was seen in the basal B MB-231 cells. Both basal B cell lines had the greatest levels of IFNγ inducible PD-L1 expression, especially with HCC38 cells. The results were very reproducible and the results shown are from the experiment run in triplicate. Representative flow data for one of the runs is provided in figure S1.

### Flow cytometry analysis of PD-L1 protein localization in the ATCC cell lines

**Figure 2:**
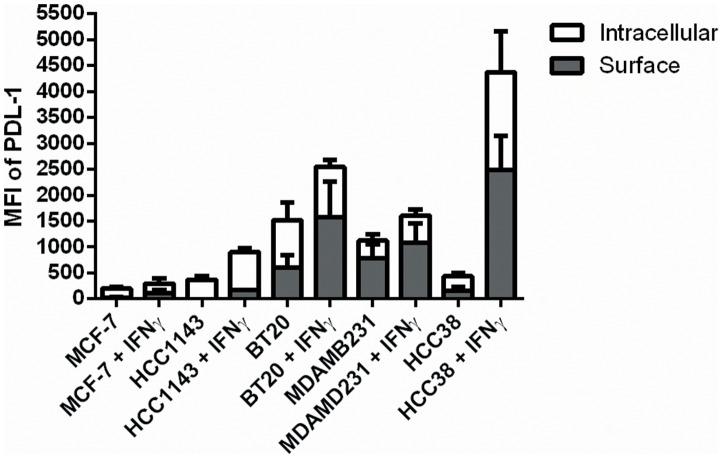
PD-L1 protein localization at baseline and post IFNγ treatment. PD-L1 protein expression increases with treatment, but there does not appear to be a major shift in protein localization patterns (proportion of surface vs. surface+intracellular) between the different cell lines and molecular subtypes.

We sought to determine if the difference in surface PD-L1 expression between the different cell lines was due to preferential localization of the protein to the cell surface or just reflective of overall increased amounts of total protein expression. Figure 2 shows the results of our flow cytometry analysis to quantify the cell surface and intracellular fractions of PD-L1 protein across the different cell lines. The same pattern of higher constitutive and inducible PD-L1 expression in the basal cell lines compared to the luminal cell lines was seen. It appears that the amount of surface PD-L1 expression in all six cell lines tested was representative of the overall amount of protein expression. Furthermore it appears that treatment with IFNγ did not result in a significant shift in the proportion of surface versus intracellular protein when compared to the constitutive PD-L1 expression for each cell line. Representative flow data for protein localization in BT20, HCC1143, HCC38, and MB-231 cell lines is provided in figures S2-1 and S2-2.

### PD-L1 mRNA expression in multiple breast cancer cell lines within the CCLE database

**Figure 3:**
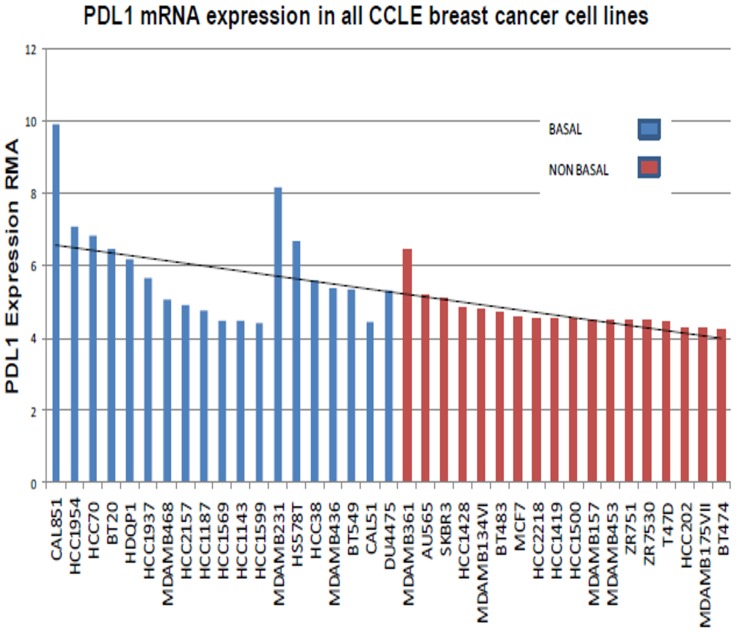
PD-L1 mRNA expression across multiple cell lines in the CCLE database. Basal cell lines as a group had statistically higher mean PD-L1 expression levels compared to non-basal subtypes.

**Figure 4:**
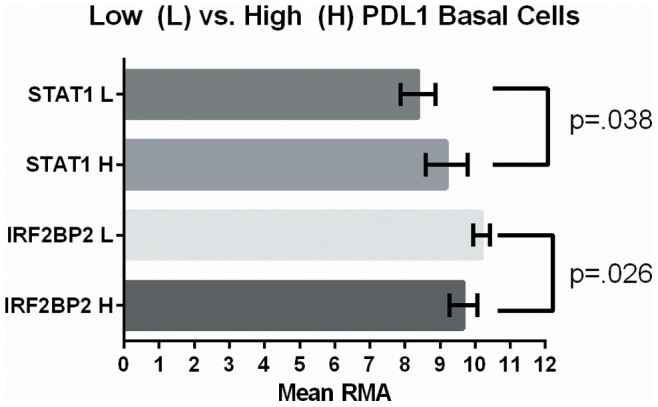
High PD-L1 expressing basal breast cancer cell lines (N = 12) demonstrate higher levels of STAT1 expression and lower levels of IRF2BP2 compared to low PD-L1 expressing cell lines (N = 12). Error bars are 95% CI.

The gene expression data for all the breast cancer cell lines within the CCLE database was studied to see if the pattern of higher PD-L1 expression in basal cell lines could be confirmed in a larger dataset. Figure 3 shows the RMA expression level of PD-L1 for basal and non-basal breast cancer cell lines. The mean RMA value of the basal cell lines was significantly greater than the non-basal group (6.37 versus 4.6, p = .002). It was noted that there were basal breast cancer cell lines that had lower expression of PD-L1 mRNA similar to the non-basal cell lines. Based on this observation, a GSEA gene analysis was run to find genes differentially expressed in high PD-L1 expressing basal cell lines as compared to the low expressing basal cell lines. Table 2 lists the pathways identified by GSEA (along with their corresponding genes) as being significantly enriched within the high PD-L1 expressing cell lines. Of particular note there were multiple pathways (MCALPAINPATHWAY, INTEGRINPATHWAY, RHOPATHWAY) involved in cytoskeleton interactions and cell motility required for invasiveness. Four pathways involved in immune mediated signaling (GATA3PATHWAY, HYPERTROPHY, and IL6PATHWAY) were found as well. Finally, genes involved in coagulation (BLOOD_CLOTTING) including serpin peptidase inhibitor, clade E member 1 (SERPINE1) and plasminogen activator, urokinase (PLAU) were enriched in the high PD-L1 group. Since PD-L1 has interferon and STAT1 response elements within its promoter we analyzed multiple mRNA levels of proteins known to regulate IFNγ induced expression in 24 basal cell lines divided into two groups of twelve based on the median PD-L1 expression level. There was a trend for higher mean STAT1 expression in high versus low PD-L1 expressing cell lines (9.19 (CI 95% = 8.59–9.79) and 8.38 (CI 95% = 7.88–8.87), p = .03). Also, mean interferon regulatory factor 2 binding protein 2 (IRF2BP2) trended lower in the high PD-L1 group compared to the low PD-L1 group of cell lines (9.66 (CI 95% = 9.26–10.07) and 10.18 (CI 95% = 9.93–10.43), p = .02). (Figure 4) IRF2 is a negative regulator of IFNγ induced gene expression, and IRF2BP2 acts as a co-repressor protein along with IRF2 to more effectively silence IFN γ induced gene expression. [11], [12] This pattern of higher STAT1/lower IRF2BP2 expression may partially explain the higher constitutive PD-L1 levels observed in some basal cell lines. Somatic mutations between the high PD-L1 and low PD-L1 basal cell lines were compared and there were no unique functional mutations or polymorphisms found in either group. This likely suggests there isn’t one dominant driver mutation which accounts for differences in PD-L1 expression between the different basal cell lines. The gene expression data file from CCLE is provided in file S1.

**Table 2:** Differentially expressed genes associated with high PD-L1 expressing basal breast cancer cell lines compared to low PD-L1 expressing basal breast cancer cell lines in CCLE GSEA analysis.

GSEA ENRICHED PATHWAYS			
AMINO ACID SUGARS METABOLISM		IL6 SIGNALING	
GNE	glucosamine (UDP-N-acetyl)-2-epimerase/N-acetylmannosamine kinase	IL6	interleukin 6
CTBS	chitobiase, di-N-acetyl-	JAK2	Janus kinase 2
HK2	hexokinase 2	JAK1	Janus kinase 1
MTMR2	myotubularin related protein 2	IL6ST	interleukin 6 signal transducer
NANS	N-acetylneuraminic acid synthase	STAT3	signal transducer and activator of transcription 3
CYB5R1	cytochrome b5 reductase 1	SHC1	SHC (Src homology 2 domain containing) transforming protein 1
HK1	hexokinase 1	MAPK3	mitogen-activated protein kinase 3
MTMR6	myotubularin related protein 6	**INTEGRIN** **PATHWAY**	
NAGK	N-acetylglucosamine kinase	CAV1	caveolin 1, caveolae protein
CMAS	cytidine monophosphateN-acetylneuraminic acid synthetase	FYN	FYN oncogene related to SRC, FGR, YES
CYB5R3	cytochrome b5 reductase 3	RAP1A	RAP1A, member of RAS oncogene family
HEXB	hexosaminidase B (beta polypeptide)	PXN	paxillin
UAP1	UDP-N-acteylglucosamine pyrophosphorylase 1	ACTA1	actin, alpha 1, skeletal muscle
**BLOOD CLOTTING**		ZYX	zyxin
SERPINE1	serpin peptidase inhibitor, clade E, member 1	ACTN1	actinin, alpha 1
SERPINB2	serpin peptidase inhibitor, clade B, member 2	BCAR1	breast cancer anti-estrogen resistance 1
PLAU	plasminogen activator, urokinase	CAPN1	calpain 1, (mu/I) large subunit
PLAT	plasminogen activator, tissue	VCL	vinculin
FGG	fibrinogen gamma chain	ROCK1	Rho-associated, coiled-coil containing protein kinase 1
**GATA3 PATHWAY**		MAP2K2	mitogen-activated protein kinase kinase 2
GATA3	GATA binding protein 3	CAPNS1	calpain, small subunit 1
PRKAR2B	protein kinase, cAMP-dependent,regulatory, type II, beta	ACTN3	actinin, alpha 3
PRKAR2A	protein kinase, cAMP-dependent,regulatory, type II, alpha	MAPK1	mitogen-activated protein kinase 1
PRKACB	protein kinase, cAMP-dependent, catalytic,beta	**MCALPAIN** **PATHWAY**	
JUNB	jun B proto-oncogene	EGFR	epidermal growth factor receptor
MAP2K3	mitogen-activated protein kinase kinase 3	CAPN2	calpain 2, (m/II) large subunit
**G COUPLED PROTEIN RECEPTORS**		**RHO PATHWAY**	
FOS	v-fos FBJ murine osteosarcoma viral oncogene homolog	ARPC1B	actin related protein 2/3 complex, subunit 1B
JUN	jun oncogene	GSN	gelsolin
PPP3CA	protein phosphatase 3, catalytic subunit, alpha isoform	ACTR3	ARP3 actin-related protein 3 homolog (yeast)
GNGT1	guanine nucleotide binding protein,gamma transducing activity polypeptide 1	ARHGEF1	Rho guanine nucleotide exchange factor (GEF) 1
MAP2K1	mitogen-activated protein kinase kinase 1	LIMK1	LIM domain kinase 1
PRKCA	protein kinase C, alpha	ARHGAP4	Rho GTPase activating protein 4
RPS6KA3	ribosomal protein S6 kinase, polypeptide 3	ARHGEF5	Rho guanine nucleotide exchange factor (GEF) 5
CALM3	calmodulin 3 (phosphorylase kinase, delta)	ARHGAP1	Rho GTPase activating protein 1
HRAS	v-Ha-ras Harvey rat sarcoma viral oncogenehomolog	TLN1	talin 1
**HYPERTROPHY**		ARPC2	actin related protein 2/3 complex, subunit 2
IL18	interleukin 18	ARHGAP5	Rho GTPase activating protein 5
CYR61	cysteine-rich, angiogenic inducer, 61	ARPC4	actin related protein 2/3 complex, subunit 4
HBEGF	heparin-binding EGF-like growth factor	DIAPH1	diaphanous homolog 1 (Drosophila)
ANKRD1	ankyrin repeat domain 1	MYL2	myosin, light chain 2, regulatory, cardiac, slow
WDR1	WD repeat domain 1	PFN1	profilin 1
DUSP14	dual specificity phosphatase 14	CFL1	cofilin 1 (non-muscle)
		MYLK	myosin, light chain kinase
		ACTR2	ARP2 actin-related protein 2 homolog (yeast)
		**THROMBOPOETIN PATHWAY**	
		STAT1	signal transducer and activator of transcription 1

Some genes were represented multiple times in different pathways so duplicate entries were deleted.

### Relationship between PD-L1 expression by mRNA, immunohistochemistry and clinicopathologic factors in patient samples

**Figure 5:**
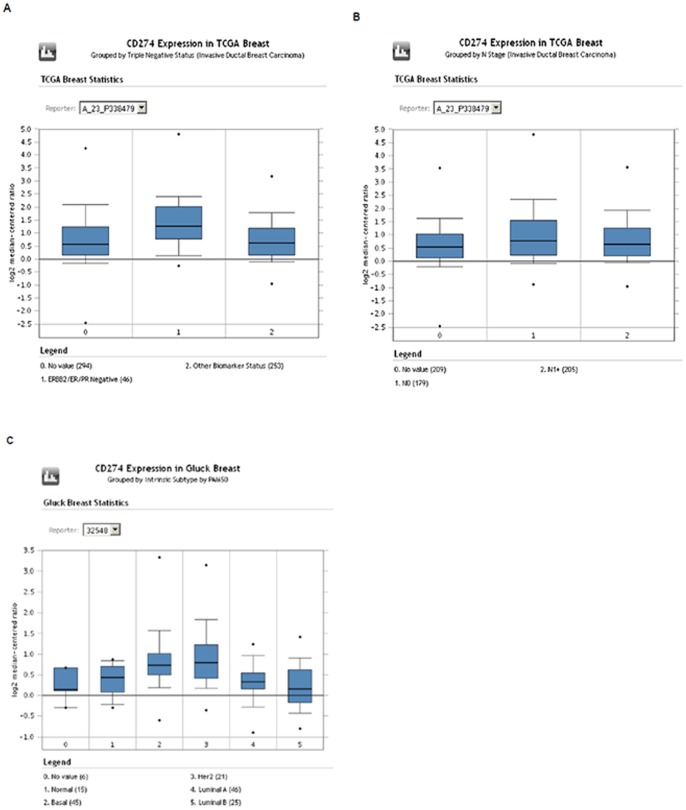
Oncomine box plot RNA expression data for PD-L1 (CD274) shown within the TCGA (Fig 5A. TNBC and Fig 5B. nodal status) and Fig 5C. Gluck datasets (PAM50 data).

The box plot data from the Oncomine analysis are shown in Figure 5. Triple negative breast cancer samples in TCGA had a higher log2 median expression compared to non-triple negative 1.28 (−.27–4.80) versus.60 (−.97–3.17). Median expression between node positive and node negative samples did not appear different. Molecular subtyping within the Gluck dataset showed lower median log2 levels in luminal A subsets (.33 (−.90–1.26)) compared to basal (.72 (−.60–3.32)).

**Figure 6:**
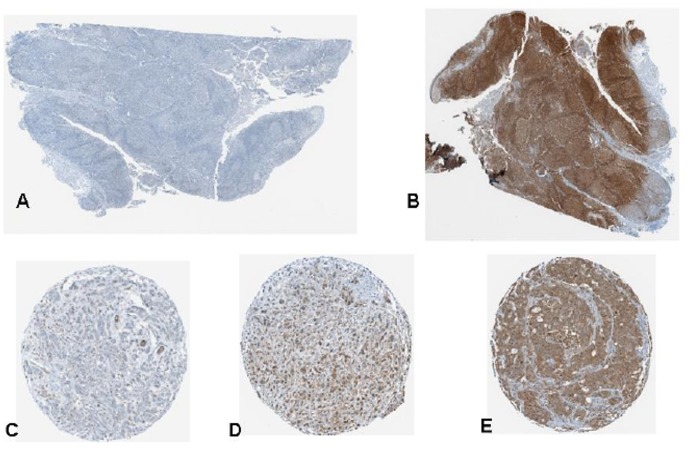
Immunohistochemistry for PD-L1 in a breast cancer TMA. A) Negative control B) Positive control C) low D) intermediate E) strong.

**Figure 7:**
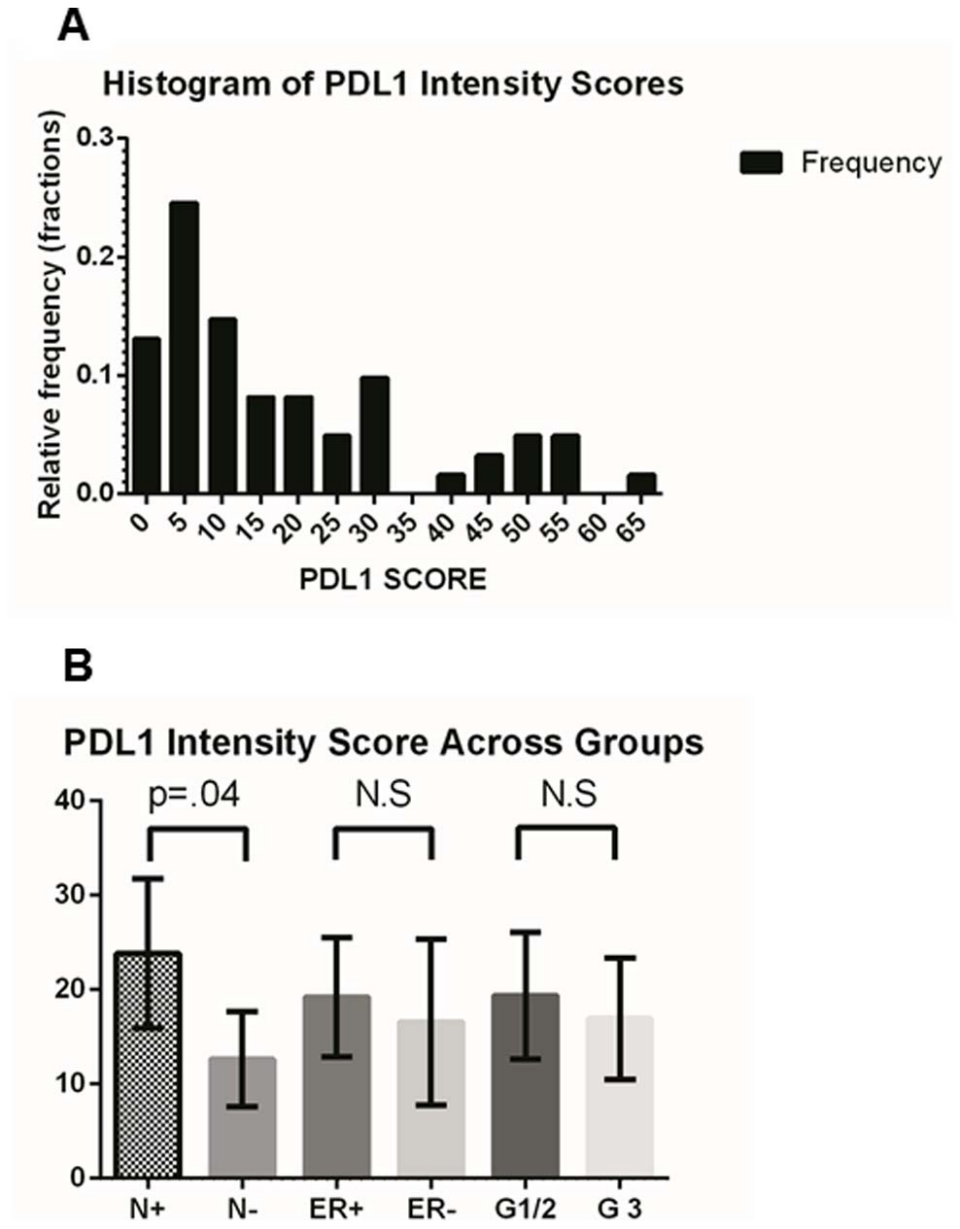
PD-L1 expression in node negative and node positive breast cancer tissue microarray samples. Figure 7a shows the distribution of PD-L1 scores across the 61 TMA samples. Figure 7b shows the mean PD-L1 expression was significantly higher in node positive (N+) compared to node negative (N−) cases overall (error bars are 95% CI).

Sixty one invasive breast cancer primary tumors were examined using immunohistochemistry for PD-L1 expression in a tissue microarray using a digital image analysis algorithm. (Figure 6) The software demonstrated an excellent ability to localize protein expression within the various cores. Concordance with pathologist scoring in terms of relative intensity (absent/low, intermediate, and high) was >90%. The distribution frequency of PD-L1 scores was mostly clustered in the low-intermediate range and is shown in Figure 7a. We were not able to find a statistically significant difference in PD-L1 expression between ER+ and ER- breast cancer primaries on our TMA in contrast to earlier published reports. However, the mean PD-L1 expression value of the node positive group trended higher (23.82, 95% CI = 15.93–31.71) than node negative breast cancers (12.62, 95% CI = 7.59–17.64) with a p = .04 (Figure 7b). There were no other statistically significant differences in PD-L1 expression level in the other clinicopathologic parameters. It should be noted though that the power of the analysis was somewhat limited due to the depletion of some of the cores in the TMA which reduced the number of samples available. This association needs to be tested further in larger annotated tissue sets.

## Discussion

The data demonstrates basal subtype cell lines generally have higher constitutive expression levels compared to luminal cell lines. Those cell lines with relatively high constitutive PD-L1 expression also appear to produce much more surface PD-L1 in response to IFNγ. The observed variation in PD-L1 surface expression between cell lines is not because of differences in cell membrane protein localization but rather due to differences in overall PD-L1 expression. The cell line data also suggests that there are intrinsic differences between different breast cancers which lead to increased PD-L1 expression on the tumor cells independent of the tumor microenvironment. The combination of higher STAT1/lower IRFBP2 gene expression levels is one such mechanism which could explain this difference. Additional investigation is ongoing to confirm this hypothesis. It should be made clear that multiple pathways besides IFNγ activated JAK/STAT signaling can affect PD-L1 expression in tumor cells. Signaling through key proliferative pathways such as MEK/ERK and PI3K/AKT can also increase PD-L1 expression. [13] Understanding which tumors have a genetic propensity to greatly up-regulate PD-L1 expression is important because the expression of PD-L1 is fluid and can vary within different regions of the same tumor over time. This tumor heterogeneity can lead to false negative PD-L1 immunohistochemistry testing if the sampled region of the tumor happened to have a temporarily lower level of PD-L1 expression at that time. Clinical trials are using PD-L1 immunohistochemistry as a predictive biomarker for PD-1 blockade, but it is clear that this marker alone is not able to identify all those who would benefit from this therapy. Identifying additional biomarkers that would predict either high constitutive or inducible PD-L1 in a tumor would aid in better selecting patients for treatment with PD-1/PD-L1 antibodies.

Analysis of the CCLE expression data supports the hypothesis that basal cells with high PD-L1 differentially express several genes implicated in invasion, chemotherapy resistance, and metastatic potential. This is especially the case with some of the INTEGRIN (CAV1, PXN, ZYX, VCL), RHO (TLN1, CFL1), and calpain1/2 genes.[14]–[21] Basal B breast cancer cell lines have been shown to be more invasive in Boyden chamber experiments, providing more evidence that high PD-L1 cell lines behave in a more invasive manner. [22] Cells that are highly proliferative, invasive, and able to effectively evade cytotoxic T cells would have a greater selective advantage and likely contribute more to the progression of breast cancer. The higher expression of PD-L1 in our TMA lymph node positive breast tumors would suggest that this is the case. While the Oncomine TGCA dataset did not show a higher median PD-L1 expression in the node positive tumors, this could have been due to factors such as differences in tumor sampling, intratumoral heterogeneity, and differences between mRNA and corresponding protein levels. Additional analysis of larger annotated breast cancer tissue sets looking at both genomic and proteomic aspects of PD-L1 expression will need to be carried out to confirm any associations with clinicopathologic factors. So PD-L1 can be thought of as a potential biomarker for highly invasive breast cancer cells within a tumor that can be targeted by the immune system via PD-L1 antibodies. While these cells are only a fraction of the total number of cells within a tumor, they likely contribute disproportionately more to the progression of metastatic disease. This means that clinical trials using PD-L1 targeting agents should be powered not only to look at objective response rates using these agents but also the impact on overall survival.

It has become very clear that malignant cells must be able to successfully evade immune surveillance in order to progress and metastasize. Tumor cells use a variety of different pathways to achieve this goal including overexpression of certain cytokines (IL-4, IL-10, TGFB), catabolic enzymes such as indoleamine 2,3 dioxygenase, and also by upregulation of surface PD-L1 expression. This work suggests that as certain breast cancer cells within a tumor increase their invasive potential, they also increase their ability to evade immune surveillance and form distant metastases. Future research will need to look at the role of PD-L1 expression in models of invasion, epithelial-mesenchymal transition, and in patients with circulating tumor cells available for analysis. Effective immune eradication of these highly invasive cells via PD-L1 antibody therapy may prove to be an effective strategy for limiting the progression of micrometastatic breast cancer.

## Conclusions

- A subset of basal type breast cancer cell lines express higher levels of PD-L1 compared to other basal and luminal breast cancer cell lines. These high expressing PD-L1 basal cells differentially express genes involved in motility, invasion, and drug resistance at a higher level compared to low PD-L1 expressing basal cell lines.
- One possible mechanism for the higher constitutive expression of PD-L1 in certain basal breast cancer cell lines is higher levels of STAT1 and lower levels of IRF2BP2 expression.
- This data suggests that immune evasion and increased metastatic potential through processes like epithelial to mesenchymal transition in tumor cells are linked. Use of PD-L1 blockade in clinical trials may selectively target these aggressive clones for immune destruction and improve outcomes.

## Supporting Information

Figure S1 **Flow cytometry data for surface expression of PD-L1 in six breast cancer cell lines +/− IFN gamma treatment for 48 hours.**
(TIF)

Figure S2 **Protein localization flow cytometry data for basal cell lines.** The fluorescence curves and MFI values shown in the legends are isotype surface and intracellular staining, then PD-L1 mAB surface+isotype intracellular staining, followed by PD-L1 mAB surface+PD-L1 mAB intracellular staining.
(TIF)

File S1 **CCLE gene expression data for cell lines.**
(GCT)
