# Supplementary figures and images for: PD-L1 Expression Is Increased in a Subset of Basal Type Breast Cancer Cells
Source: PLoS One. 2014 Feb 14;9(2):e88557. doi: 10.1371/journal.pone.0088557 (PMC3925108; doi:10.1371/journal.pone.0088557)

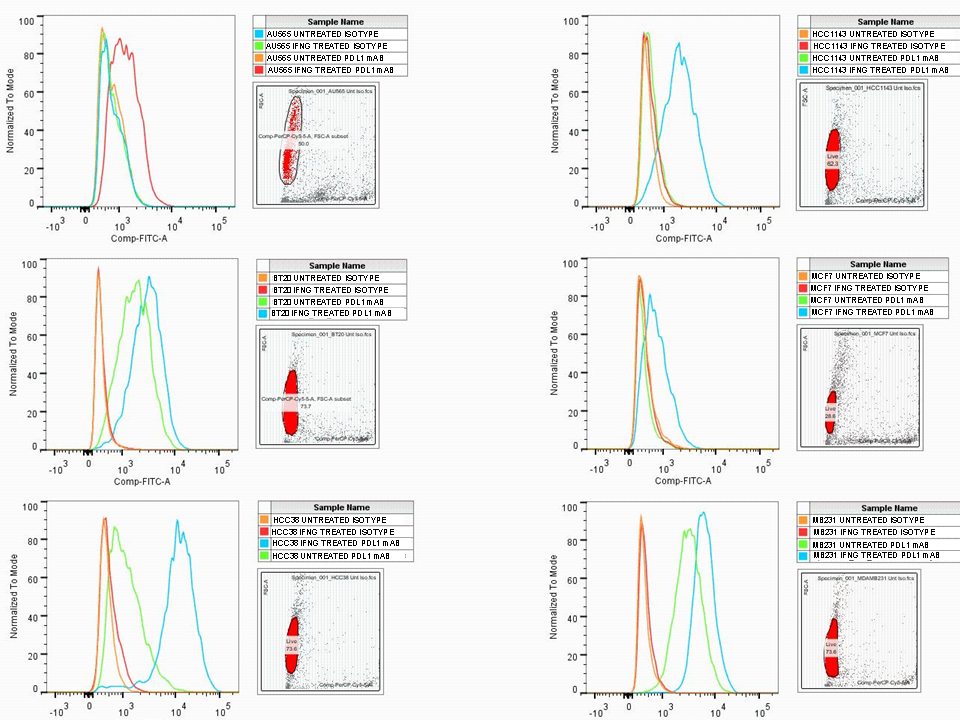

Supplement: Figure S1 — Flow cytometry data for surface expression of PD-L1 in six breast cancer cell lines +/− IFN gamma treatment for 48 hours. (TIF) [file pone.0088557.s001.tif]

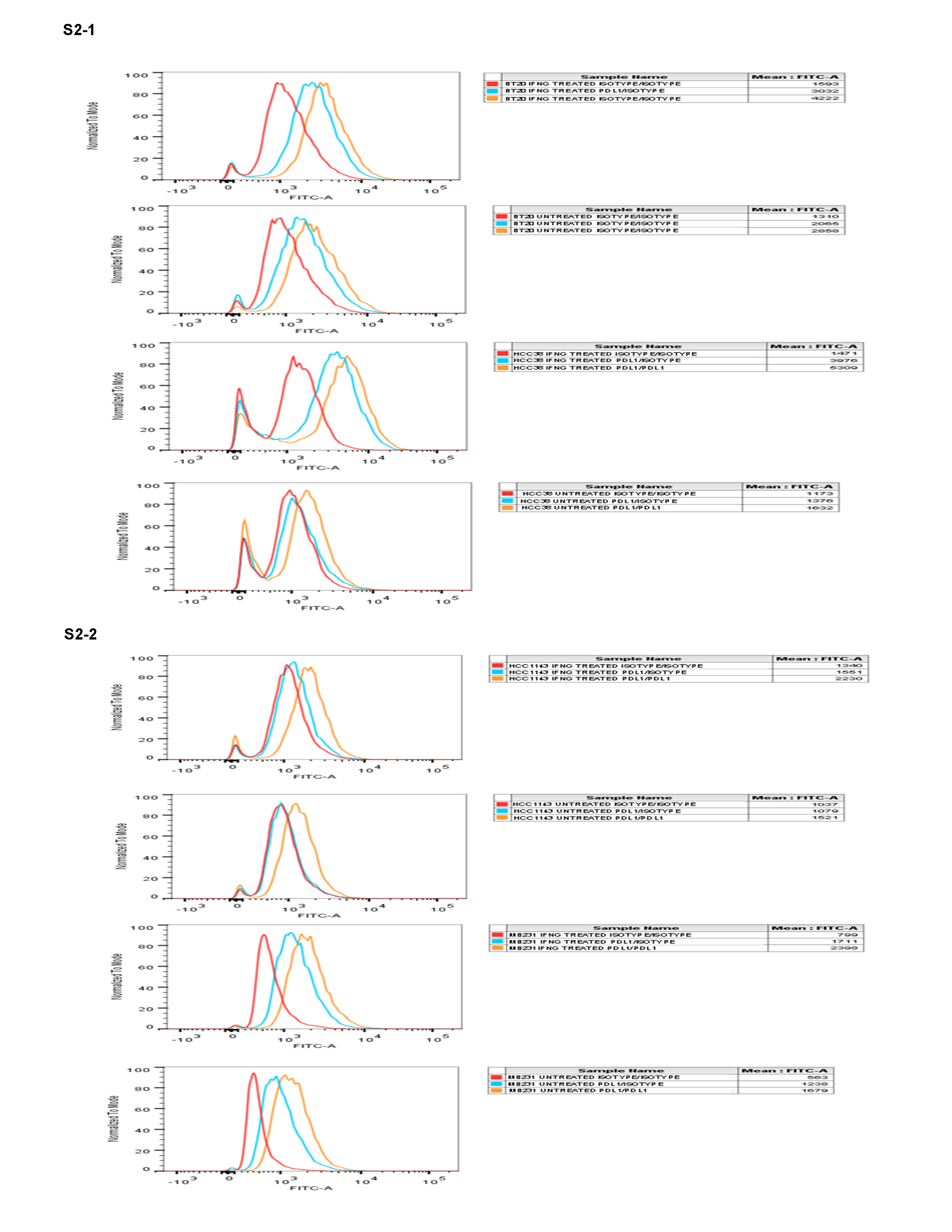

Supplement: Figure S2 — Protein localization flow cytometry data for basal cell lines. The fluorescence curves and MFI values shown in the legends are isotype surface and intracellular staining, then PD-L1 mAB surface+isotype intracellular staining, followed by PD-L1 mAB surface+PD-L1 mAB intracellular staining. (TIF) [file pone.0088557.s002.tif]
